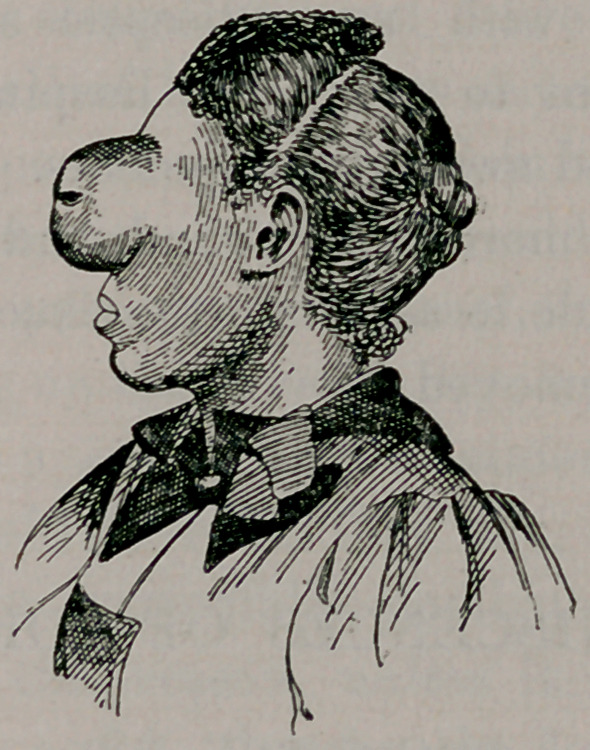# Amputation of Breast: Tumor of Orbit: Supernumerary Digit: Gunshot Wound of Ankle*Clinical lecture delivered at Southern Medical College, Atlanta, Ga., March 12th, 1898. Reported by Dr. Claude A. Smith.

**Published:** 1898-11

**Authors:** Wm. Simpson Elkin

**Affiliations:** Professor Operative and Clinical Surgery, Atlanta College of Physicians and Surgeons, Atlanta, Ga.


ATLANTA

### Medical and Surgical Journal.

Vol. XV. NOVEMBER, 1898. No. 9.

DUNBAR ROY, A.B., M.D., EDITOR.

M. B. HUTCHINS, M.D., BUSINESS MANAGER.

### ORIGINAL COMMUNICATIONS.

### AMPUTATION OF BREAST: TUMOR OF ORBIT: SUPERNUMERARY DIGIT: GUNSHOT WOUND OF ANKLE.*

By WM. SIMPSON ELKIN, M.D.,

Professor Operative and Clinical Surgery, Atlanta College of Physicians and Surgeons, Atlanta, Ga.

Our first patient is a negro woman, aged twenty-three years, single, washerwoman, family and personal history negative.

About three years ago she noticed a small lump or nodule in the upper and outer quadrant of the left breast, which has gradually grown until it has attained its present size—that of a large orange. The tumor is circumscribed, firm and elastic, freely movable. The skin is not adherent to the growth. The nipple is not retracted. No enlargement of the axillary glands, and no pain. I believe we have in this case an adeno-fibroma, and I have advised its removal. Adeno-fibromata are the most frequent benign mammary neoplasms, and when removed do not recur.

* Clinical lecture delivered at Southern Medical College, Atlanta, Ga., March 12th, 1898. Reported by Dr. Claude A. Smith.

They consist of hypertrophied connective tissue mixed with more or less glandular structure, and differ only from the hypertrophied mamma by being circumscribed and encapsulated.

If this were a malignant tumor you would have a history of a more rapid development, pain, hard nodular mass more or less movable, adherent skin, retracted nipple, and at this time an involvement of the adjacent axillary glands. All tumors of the breast, whether benign or malignant, should be removed as early as possible, since benign tumors frequently assume a malignant character. It is not always necessary to sacrifice the whole breast, and I would not do so in this instance if the tumor was smaller, and did not occupy so much of the gland. Smaller tumors of this character may be dissected out, and the usefulness of the breast retained.

The mammary gland of the female is situated between the third and fifth ribs, and is supplied from the inner side by the second, third, fourth and fifth intercostal branches of the internal mammary artery, and on the outer side by the acromio-thoracic, long thoracic and external mammary arteries. The long thoracic and external mammary are the largest arteries that supply the breast, and consequently give more hemorrhage when divided.

Although the seat of this operation was thoroughly sterilized on yesterday afternoon, and has since been enveloped in an antiseptic dressing, I will have the nurse scrub it again with tincture of green soap and water, and then wash with 1 to 1,000 bichloride solution, and lastly with alcohol or ether.

The bowels have been moved by a saline cathartic, and the patient instructed to eat a light breakfast. Anesthesia being now complete, the patient is drawn near the edge of the table. Shoulders are slightly elevated by placing a pillow or sandbag beneath them. The arm of the affected side is raised, and the hand put beneath the head. The operator stands on the affected side, and faces the patient if the right side is involved, and faces the lower extremity of the opposite side if the left breast is to be removed.

In amputations of the breast the incision is an elliptical one, the middle of the ellipse being opposite the nipple.

As this is the left breast, I begin my incision on the lower and inner side, carrying it over the gland parallel to the lower fold of the pectoralis major muscle, terminating it well above the upper and outer margin of the breast. A similar incision is made in the under surface of the mamma, beginning and terminating at the same points, thus completing the ellipse. This will give us sufficient skin to cover the surface after the breast is removed. The incision should be completed at once, and is carried through the skin and superficial fascia. We stop at this point to check the hemorrhage. I now pull the breast downward, while my assistant retracts the skin upward and inward, and divides all the tissues down to the pectoralis major muscle. The dissection is carried downward, and the mamma is raised from the muscles beneath. As the hemorrhage is quite extensive we will apply the compression forceps and ligate these arteries before proceeding further. The breast, you now see, is dissected well up from the chest wall, and all we have to do is to extend the lower incision through the fascia down to the muscles, completing the incision at the upper and outer border below the fold of the pectoralis major muscle. As the large blood-vessels enter the gland at this point I divide them last. You see now that the edges of the incision can be easily approximated, and we have a straight line of closure. The hemorrhage is well checked, the arm brought down to the side of the chest, and the wound is closed by the introduction of interrupted silkworm-gut sutures. No drainage is necessary. A strip of iodoform gauze is placed over the line of incision. Over this we place sterile and bichloride gauze, absorbent cotton, and lastly a roller bandage is applied firmly over this dressing. The pressure of the bandage firmly applied stops any oozing. It is necessary, by a few turns of the bandage, to secure the arm of the affected side to the chest wall, otherwise the movements of the pectoral muscles would disturb the healing of the wound.

If the temperature remains practically normal, the dressings will not be changed until seven or eight days, when they will be taken off and the stitches removed. A light dressing will then be applied, and in two weeks the patient should be entirely well.

The next case is very unique. It is a growth involving the left orbit.

Mattie Ward, aged eighteen years, single, black, female, washerwoman.

Grandparents all dead—cause unknown. Two or three brothers and sisters died when very small—cause of death not known. Father and mother alive and well at about forty years. One brother and three sisters living, all in good health—ages three to twenty-five years.

No history of syphilis or gonorrhea obtained. Has had pneumonia two years ago, and whooping-cough at five years of age. No history of inflammation of eye previous to beginning of this trouble obtainable. Dates trouble from time of whooping-cough, and states this was the cause. During a paroxysm of coughing, she says, her eye "popped out." Four years ago, when she left her mother, eye was closed and beginning to bulge some, but not very much. Has been slowly growing since she can remember. Has about doubled in last two years, and appears to be growing more rapidly now than formerly. At times she has severe neuralgia above eye, and general headaches, which she does not seem to be able to separate intelligently. She sheds tears and winks this eye with the other. It is quite sensitive, so much so that examination is not satisfactory. Tumor is cystic or semi-cystic, and rounded. Somewhat larger than a baseball. Skin seems normal except for some large veins visible. Bony wall is bulged out in all directions. Nose turned to opposite side. The growth bulges forward about two and one-half inches. Measures ten inches in circumference and three in diameter, and has the feel of a cystic or semi-solid tumor. The orbital border is large and flared out, the nasal bone pushed to the opposite side and the molar bone downward. The edges of the lids have grown together, and it presents the condition of ankyloblepharon. Ankyloblepharon is not a common occurrence, usually exists in children, and may be partial or complete. This tumor may be the result of accumulation of tears and mucus behind the lids, the eyeball being forced back into the orbit. Doctors Calhoun, Stirling and Roy of this city have each examined this patient and testify to the unique and unusual appearance of the growth. They are inclined to the opinion of malignancy.

I will make an incision in the line of the palpebral fissure, separating the upper and lower lids. The lashes of the lower lid are absent. After making this incision you observe the round and shining appearance of the growth, which I take to be its capsule. On opening this I am able to liberate a mass of tissue resembling very much in appearance a sarcoma. A careful microscopical examination will be made to determine the exact character. I was surprised not to find more fluid, as it gave evidence of being more or less cystic. I will now remove the capsule and other affected tissue if possible, check the hemorrhage, and pack the orbit with iodoform gauze, and apply external antiseptic dressing. In a few days I hope to be able to give you the result of the microscopical examination.

(The accompanying cut shows appearance of tumor before removal. It proved to be a myxosarcoma. The patient made a prompt recovery and to-day, six months after operation, is perfectly well.)

The third case that I present is a young boy, aged four years. He has two thumbs on the same hand. He has what is known as a supernumerary digit. This condition may affect not only the thumb and fingers, but also the toes, most usually involving the thumb and little finger, and the small toe. The supernumerary digit may be attached by a small, narrow, fibrous band, or may have its own phalanx, and sometimes a separate metacarpal or metatarsal bone. They are most usually inherited and should be removed as early as possible. The operation is very simple, and consists in making an incision around the base of the supernumerary digit, and amputating as you would a finger or toe. If the patient is young, there is very little hemorrhage, and the incision heals readily.

The next and last case is a negro man, aged forty-two years, married, carpenter. He states that he was shot night before last, the ball entering the leg on the inner side of the tendo Achillis, just above and behind the internal malleolus, passing downward and lodging in the tarsal or metatarsal bone of the foot. He does not know the size of the ball, but states that he was shot from behind at a distance of about thirty feet. It would be unsurgical to probe for this ball, and I would only direct that the wound of entrance be thoroughly cleansed with an antiseptic solution, and dressed, instructing the patient to go to the hospital and remain in bed with foot elevated, and await developments. If suppuration takes place, the ball can be more easily found, and then can be removed. An X-ray may be made to ascertain the exact location of the ball, which may then be removed.

## Figures and Tables

**Figure f1:**